# Proving of Syphilinum

**Published:** 1882-02

**Authors:** E. W. Berridge

**Affiliations:** London


### PROVING OF SYPHILINUM.

E. W. Berridge, M. D., London.

In my paper on "The Scientific Use of the Nosodes," I endeavored to demonstrate, both by logical argument and by practical experience, that to give a nosode, indiscriminately in *all* cases of the corresponding disease, was unscientific in theory and unsatisfactory in practice; adducing as a reason the fact that diseases were so frequently complicated, and that the nosode would fail to *cure*, unless it corresponded to the totality of the symptoms, according to the law of *similars.* As a necessary deduction from this argument, it follows that we must obtain an accurate and thoroughly reliable pathogenesis of the nosodes, which may be supplemented and aided by careful clinical experience, before we can prescribe them strictly according to the totality of the symptoms; and without this our success, though at times it may be great, will always be uncertain.

As a preparatory contribution to the particular forms of syphilis, in which *Syphilinum* is indicated, I copy the following proving from Dr. Swan's pathogenesis thereof, a copy of which lies before me:

"A gentleman, about 60 years of age, who had no sexual desire or power for several years, had taken *Syphilinum* 200 without obtaining any symptoms therefrom. He now commenced with the 50 M (Swan), taking a pellet (No. 30) every hour, as near as possible, until he had finished the contents of a No. 1548 vial, such as Dr. Swan uses for his potencies. The proving was commenced about the last of October or first of November. Having finished the contents of the vial, and noting no symptom, he forgot all about it, till about January 2d, when he found an ulcer, the size of a split pea, on the prepuce above the corona glandis; the edges were red and raised, while the bottom was covered with a lardaceous deposit; there was no pain or sensation in it; the glans looked purple, and on the left side it was covered with an exudation. It was examined by a skillful surgeon of New York, who pronounced it to be a chancre, and the exudation on the glans decidedly diphtheritic. The prover, wishing to get rid of such a disagreeoble symptom, took a dose of MM, and by January 7th, the whole lesion had nearly disappeared."

The diphtheritic exudation on the glans is remarkable, and shows a relationship with *Lac Caninum*, which has not only cured certain cases of syphilis, when indicated by the symptoms, but also produced and cured diphtheritic membranes on the fauces. If any physician, with a case of diphtheria, with general aggravation in afternoon, evening or night, *and relief at daybreak*, let him not forget *Syphilinum.*

